# MRI Features of Intracranial Anaplastic Ependymomas: A Comparison of Supratentorial and Infratentorial Lesions

**DOI:** 10.3389/fonc.2020.01063

**Published:** 2020-07-21

**Authors:** Xin-Ping Kuai, Sheng-Yu Wang, Yi-Ping Lu, Ji Xiong, Dao-Ying Geng, Bo Yin

**Affiliations:** ^1^Department of Radiology, Huashan Hospital, Fudan University, Shanghai, China; ^2^Department of Radiology, Ruijin Hospital North, Shanghai Jiao Tong University School of Medicine, Shanghai, China; ^3^Department of Pathology, Huashan Hospital, Fudan University, Shanghai, China

**Keywords:** anaplastic ependymomas, intracranial, supratentorial, infratentorial, magnetic resonance imaging

## Abstract

**Background:** Several previous reports of anaplastic ependymomas have described their imaging features, and most of these studies were case reports. However, no studies have compared the magnetic resonance imaging (MRI) features between the infratentorial and supratentorial anaplastic ependymomas.

**Objective:** The goal of this study was to explore MRI characteristics for intracranial anaplastic ependymomas.

**Material and Methods:** We retrospectively reviewed the demographics of 165 patients and MRI findings of 60 patients with supratentorial (SAEs) and infratentorial anaplastic ependymomas (IAEs) before surgery. The demographics and MRI features for SAEs and IAEs were compared and evaluated.

**Results:** Among the 60 patients, most SAEs (91.7%) were extraventricular, whereas most IAEs (91.7%) were intraventricular. Of sixty intracranial anaplastic ependymomas, most lesions were well-defined (*n* = 45) and round-like (*n* = 36). On T1-weighted imaging, compared with the gray matter, the SAEs exhibited heterogeneous signal intensity, whereas IAEs exhibited iso-hypointense signals. T2 signals exhibited greater associations with hyperintense signals in IAEs; however, SAEs showed hyperintense or hypointense–hyperintense. On diffusion-weighted imaging (DWI), almost all solid tissues of SAEs appeared as hyperintense, whereas IAEs exhibited iso-hypointense signals. Peritumoral edema and intratumoral hemorrhage occurred more frequently in SAEs. Almost all anaplastic ependymomas exhibited heterogeneous enhancement. Cysts or necrosis was associated with 56 anaplastic ependymomas; however, large cysts were more prevalent in SAEs. On magnetic resonance spectroscopy (MRS), the mean choline/creatine (Cho/Cr) and choline/N-acetyl-aspartate (Cho/NAA) ratio of anaplastic ependymomas were (6.58 ± 4.26) and (8.84 ± 6.34), respectively, representing typical high-grade tumors.

**Conclusion:** We demonstrate the conventional and functional MRI features of intracranial anaplastic ependymomas, including DWI and MRS. MRI characteristics, such as location, cyst, diffusion restriction, and peritumoral edema, differed between supratentorial and infratentorial locations. Cho/Cr and Cho/ NAA ratios of anaplastic ependymomas are increased.

## Introduction

Ependymomas, which are central nervous system (CNS) tumors, are commonly found in the fourth ventricle and arise from ependymal cells lining the ventricular system (1). Moreover, these tumors can occur anywhere within and even outside the CNS (2–4). Based on the World Health Organization (WHO) 2016 classification system, ependymomas are divided into three histological grades: grade I–III (5). Anaplastic ependymoma has been recognized as a high-grade malignant neoplasm of the CNS and is classified as WHO grade III. Given that studies of anaplastic ependymoma are limited by its low incidence, the accuracy of preoperative diagnosis is very low. Several previous reports of anaplastic ependymomas have described their imaging features (2, 6–8), and most of these studies were case reports. Similar to grade II ependymomas, intracranial anaplastic ependymomas are also located infratentorially or supratentorially. However, no studies have compared the MRI features between the infratentorial and supratentorial anaplastic ependymomas.

Therefore, the objective of this study was to retrospectively investigate MRI characteristics of anaplastic ependymomas identified in supratentorial and infratentorial locations and to compare the age and sex distribution for each typical location. As far as we know, we conducted one of the largest series of anaplastic ependymomas published to date.

## Materials and Methods

### Patients

This retrospective study was approved by our Institutional Review Board. Informed consent was waived from all individual participants included in the study. From January 2006 to April 2020, we retrospectively identified 181 patients with a diagnosis of anaplastic ependymomas based on histopathological criteria and the WHO grading system via a computerized search of the pathological records of our hospital. Among these patients, 16 were excluded due to spinal anaplastic ependymomas. Pathologists at our institution assessed all of the pathology specimens. A total of 165 intracranial anaplastic ependymomas cases were enrolled in this study. To reduce bias, the demographics, including age and sex, were based on these 165 cases. However, 60 patients were both histopathologically confirmed and underwent preoperative MRI in our hospital.

### MR Imaging

MRI examinations were performed in 60 cases using a 3.0-T scanner (Signa; G.E., Milwaukee, MI, USA and Siemens, Verio, Germany) with an eight-channel head coil. Patients were arranged in the magnet field in a supine position. MRI protocol contained the following sequences and parameters: sagittal and axial, T1-weighted images [repetition time (TR) at 400 ms and echo time (TE) at 15 ms]; axial, T2-weighted fast spin-echo images (TR at 3,000 ms and TE at 119 ms); and fluid-attenuated inversion recovery (FLAIR; TR at 8,500 ms and TE at 138 ms). Forty-seven patients, including 40 patients with supratentorial anaplastic ependymomas (SAEs) and 7 patients with infratentorial anaplastic ependymomas (IAEs), were subject to echo-planar diffusion-weighted imaging (DWI) with three orthogonal diffusion gradients of *b* = 1,000 s/mm^2^ and one acquisition with *b* = 0 s/mm^2^ [TR/TE = 4,800/68.6 ms; matrix size = 128 × 128; field of view (FOV) = 240 × 240 mm; number of excitations (NEX) was 4; slice thickness and gap were 6 and 2 mm, respectively]. Sixty patients underwent contrast-enhanced MRI examination that included sagittal and axial T1-weighted images. A bolus of gadopentetate dimeglumine (Magnevist, Germany) was administered intravenously at a rate of 1.5–2.0 ml/s with a dose of 0.1 mmol/kg body weight. Twenty-one patients, including 20 patients with SAEs and 1 patients with IAE, were subject to multiple-voxel ^1^H-spectroscopy with the parameters of TR/TE = 1,500/144 ms; flip angle = 90; voxel size = 10 × 10 × 10 mm; and total acquisition time = 330 s.

### Image Analysis

Anonymized images were analyzed through picture archive and communication system (PACS). Two neuroradiologists (one with 15 years of experience in neuro-MR imaging and the other with 5 years of experience in neuro-MR imaging) who were blinded to the histopathological result and clinical information reviewed the MR images. Interobserver agreement for MRI features for 60 patients was good. For settling possible inconsistency and lessening intraobserver variability, the third neuroradiologist (30 years of experience in neuro-MR imaging) assessed conflictive items, and the majority opinion was accepted for analysis. The following MR features were analyzed: the maximum diameter, location, morphology, lesion margin, T1 and T2 signal intensity, DWI intensity, peritumoral edema, and the enhancement pattern. Moreover, intratumoral hemorrhage (defined as amorphous fluid that was hyperintense on T1WI), cyst or necroses (defined as hyperintense on T2WI and non-enhanced areas), and cyst size (cyst diameter >1 cm was defined as “large;” otherwise, the cyst was defined as “small”) were also analyzed.

The longest diameter of tumors were measured. Lesion sites were divided into infratentorial, supratentorial, intraventricular, and extraventricular. A well-circumscribed tumor was defined as a clear boundary of tumor obtained on contrast-enhanced MRI. The enhancement pattern was analyzed qualitatively as non-enhancement or homogeneous or heterogeneous enhancement. The peritumoral edema was categorized as absent, mild (influencing region less than a half of the lesion), moderate (influencing region greater than a half but less than that of the lesion), or marked (influencing area greater than that of lesion) (9).

On magnetic resonance spectroscopy (MRS), metabolites of non-necrotic tumor were *N*-acetyl-aspartate (NAA), creatine (Cr), and choline (Cho). The relative quantification of these metabolites was based on peak areas using curve fitting software, which was provided by the manufacturer of MRI scanner. After baseline correction, the NAA peak was assigned at 2.02 ppm, Cr at 3.02 ppm, and Cho at 3.2 ppm. In order to obtain signal integrals, automatic curve-fitting procedures were applied. Using NAA, Cr, and Cho metabolite amplitudes, metabolite ratios, including Cho/Cr and Cho/NAA, were calculated.

### Statistical Analysis

The sample was described with descriptive statistics, containing means, medians, frequencies, and ranges. In contrast, an independent two-sample *t*-test was used between the continuous data of the SAE and IAE. A chi-square test or Fisher's exact test was used to compare patients' categorical variables for SAE and IAE. The relationship of tumor diameter and peritumoral edema was analyzed using Spearman rank correlation. Analyses of data were performed by IBM SPSS Statistics 19.0 (IBM, Unite States). A *P* < 0.05 was considered as significant difference.

## Results

A total of 165 intracranial anaplastic ependymomas cases were enrolled in this study. A majority of intracranial anaplastic ependymomas (66.3%) occurred in adults (age > 18 years old). The patients' age ranged from 3 to 76 years old at diagnosis (median, 29 years old and mean, 30 years old). The male/female ratio was 1.5:1 (99:66).

### Age and Gender Distribution for Each Location

The age and gender distribution for each location are summarized in Table 1. The number of male patients with SAE or IAE was also increased compared with female patients. No significant difference in age and sex ratio were noted between SAE and IAE patients.

**Table 1:** Age and gender distribution for each location.

**Characteristic**	**Supratentorial (*n* = 105)**	**Infratentorial (*n* = 60)**	***P-*value**
Sex no. (%)			0.322
Male	66 (62.9)	33 (55)	
Female	39 (37.1)	27 (45)	
Age at diagnosis, year			0.78
Median	29	26	
Range	3–76	3–67	

### Conventional MRI and DWI Features of Anaplastic Ependymomas

**Figure 1:**
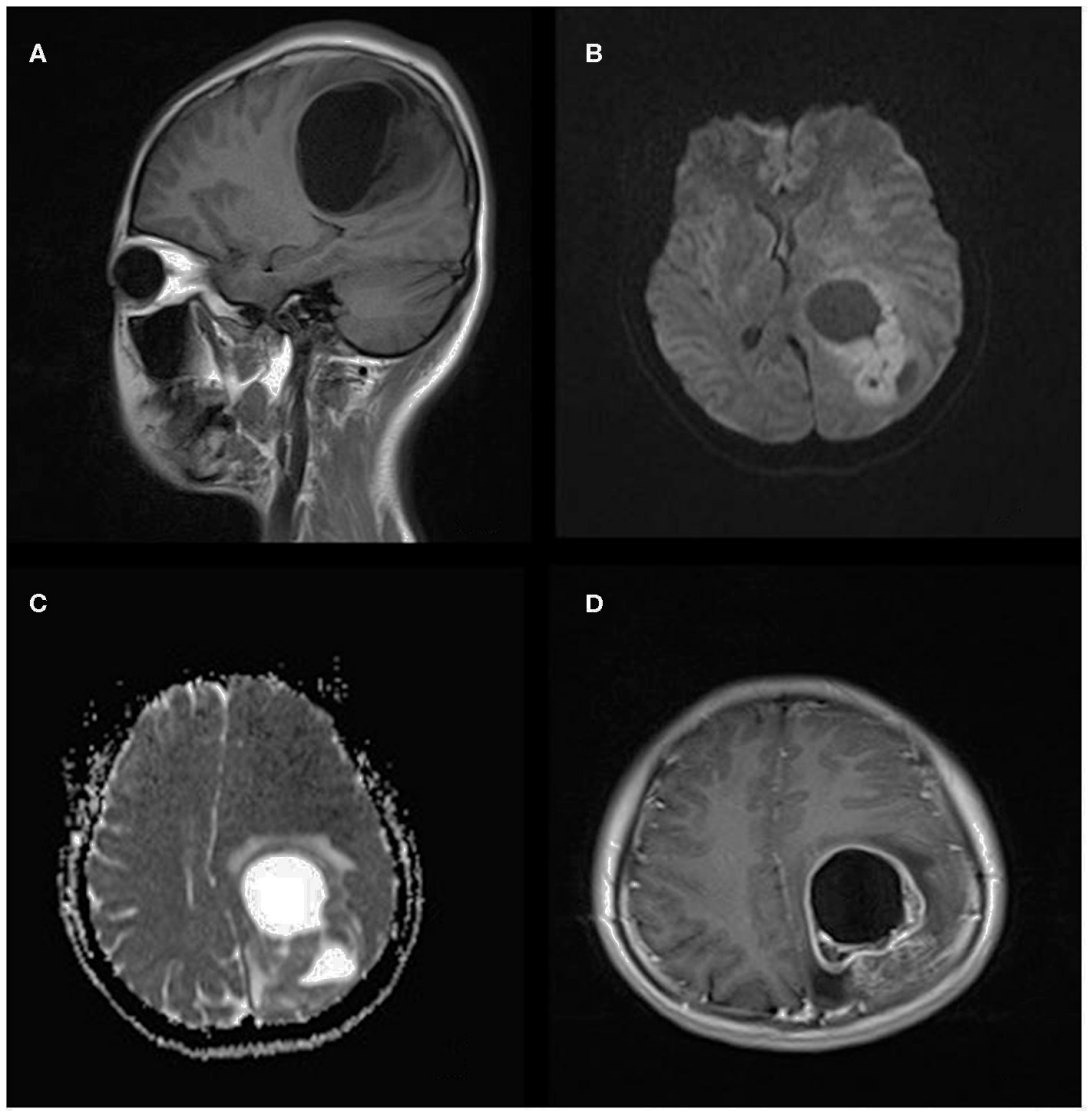
A 21-year-old woman with supratentorial intraparenchymal anaplastic ependymoma in the left parietal lobe on **(A)** T1-weighted image, **(B)** diffusion-weighted images, **(C)** apparent diffusion coefficient, and **(D)** contrast T1-weighted image. The tumor appears as heterogeneous signal intensity, large cysts, reduced diffusion within the soft tissue component, and mild peritumoral edema.

**Figure 2:**
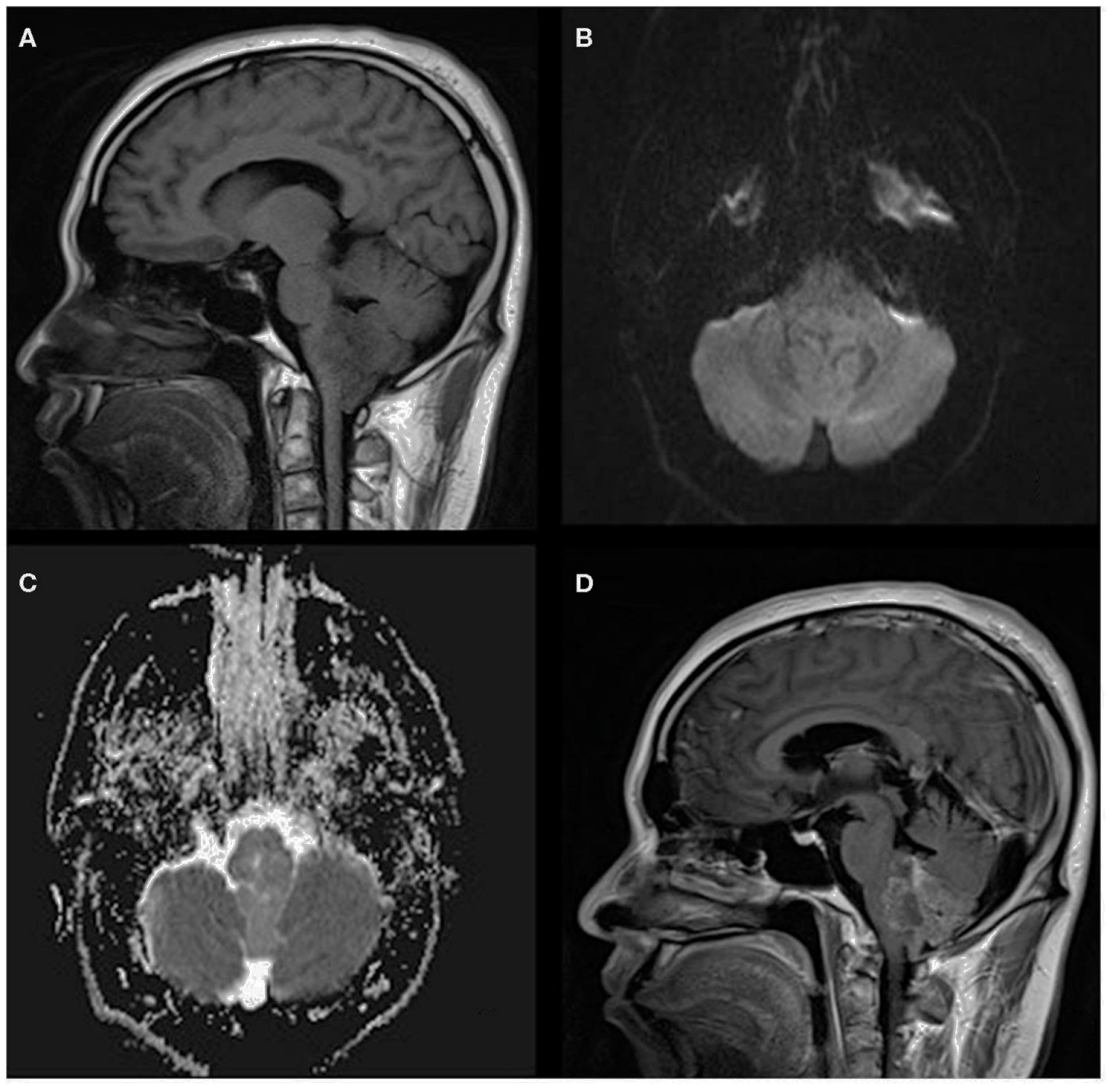
A 33-year-old man with infratentorial anaplastic ependymoma in the fourth ventricle on **(A)** T1-weighted image, **(B)** diffusion-weighted images, **(C)** apparent diffusion coefficient, and **(D)** contrast T1-weighted image. The tumor appears as heterogeneous signal intensity, small cysts, non-reduced diffusion within the soft tissue component, and absent peritumoral edema.

**Figure 3:**
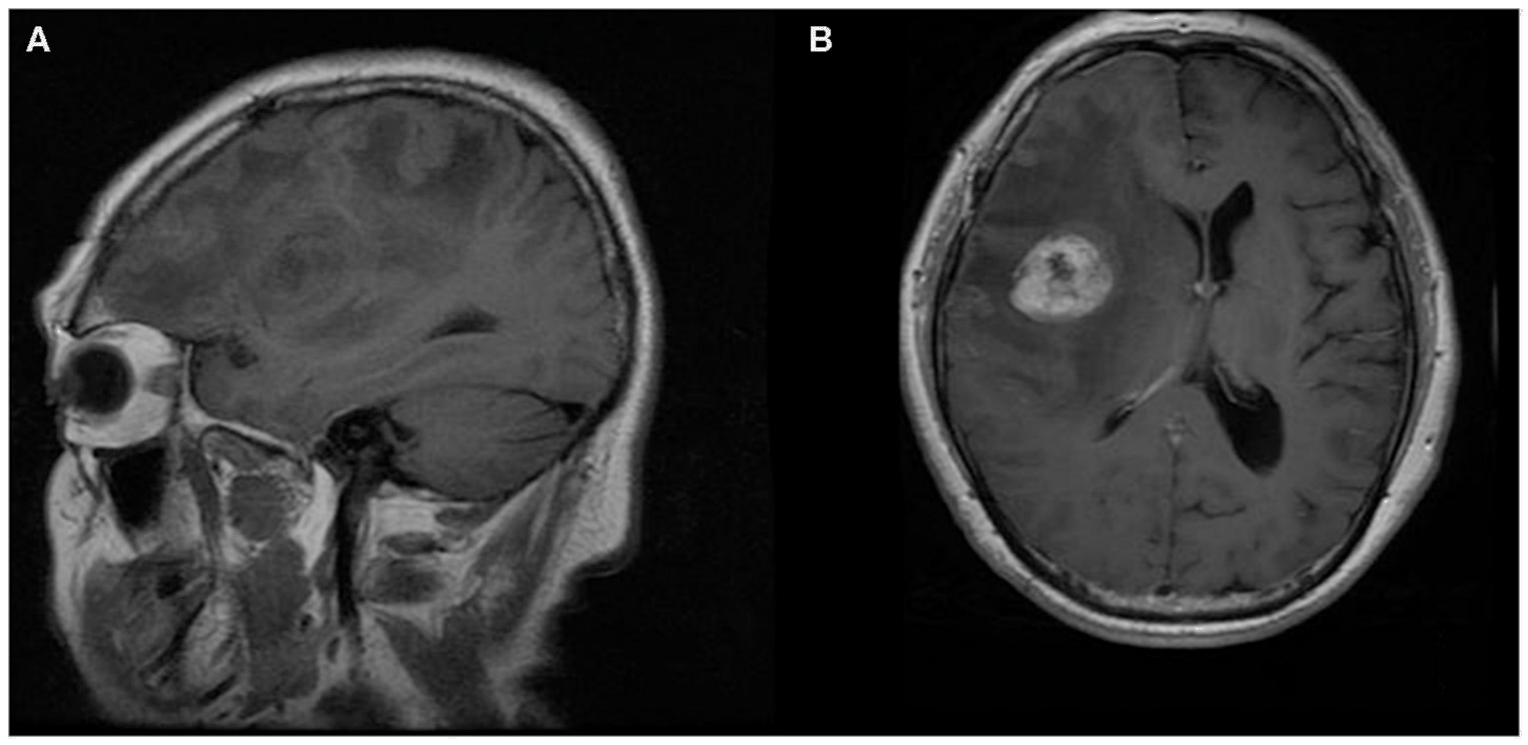
A 70-year-old man with supratentorial intraparenchymal anaplastic ependymoma in the right frontal lobe on **(A)** T1-weighted image and **(B)** contrast T1-weighted image. The tumor appears as heterogeneous signal intensity and marked peritumoral edema.

**Figure 4:**
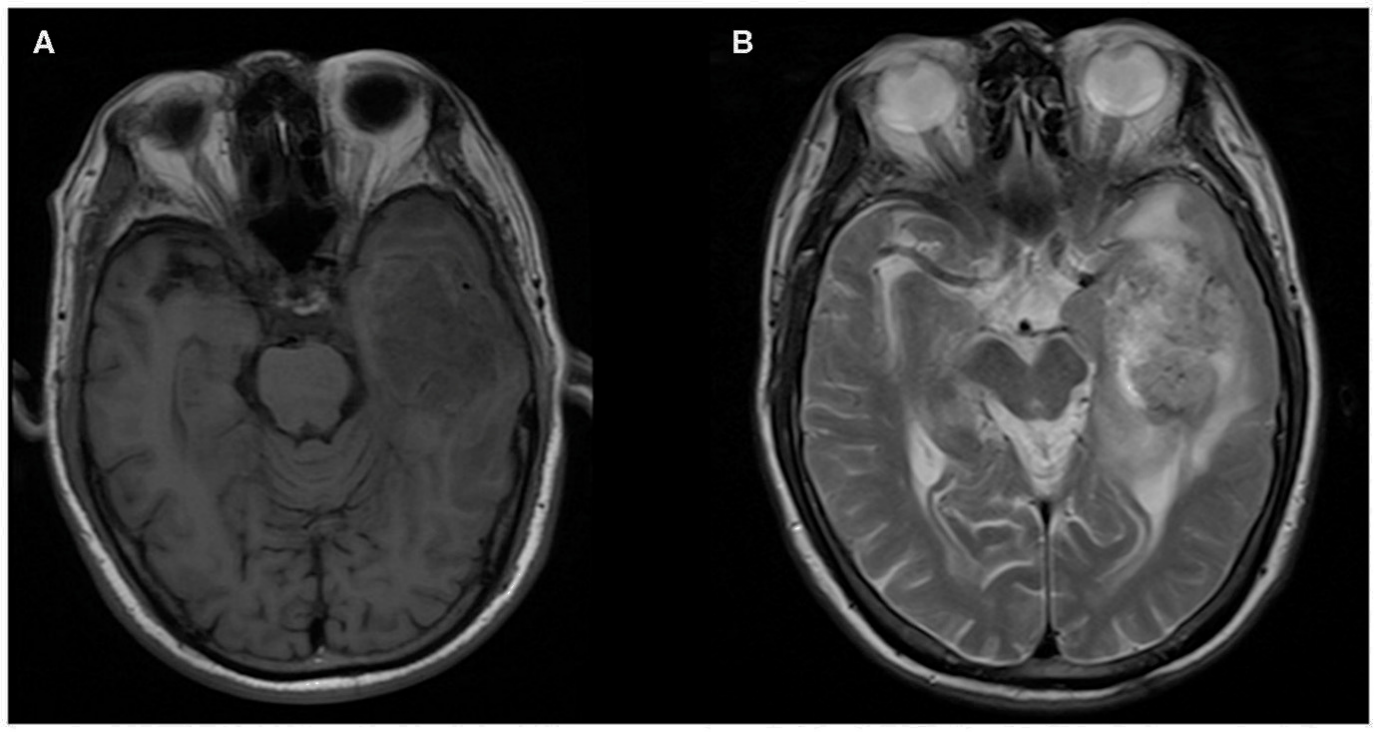
A 76-year-old man with supratentorial intraparenchymal anaplastic ependymoma in the left temporal lobe on **(A)** T1-weighted image and **(B)** T2-weighted image. The tumor appears as heterogeneous signal intensity and moderate peritumoral edema.

**Figure 5:**
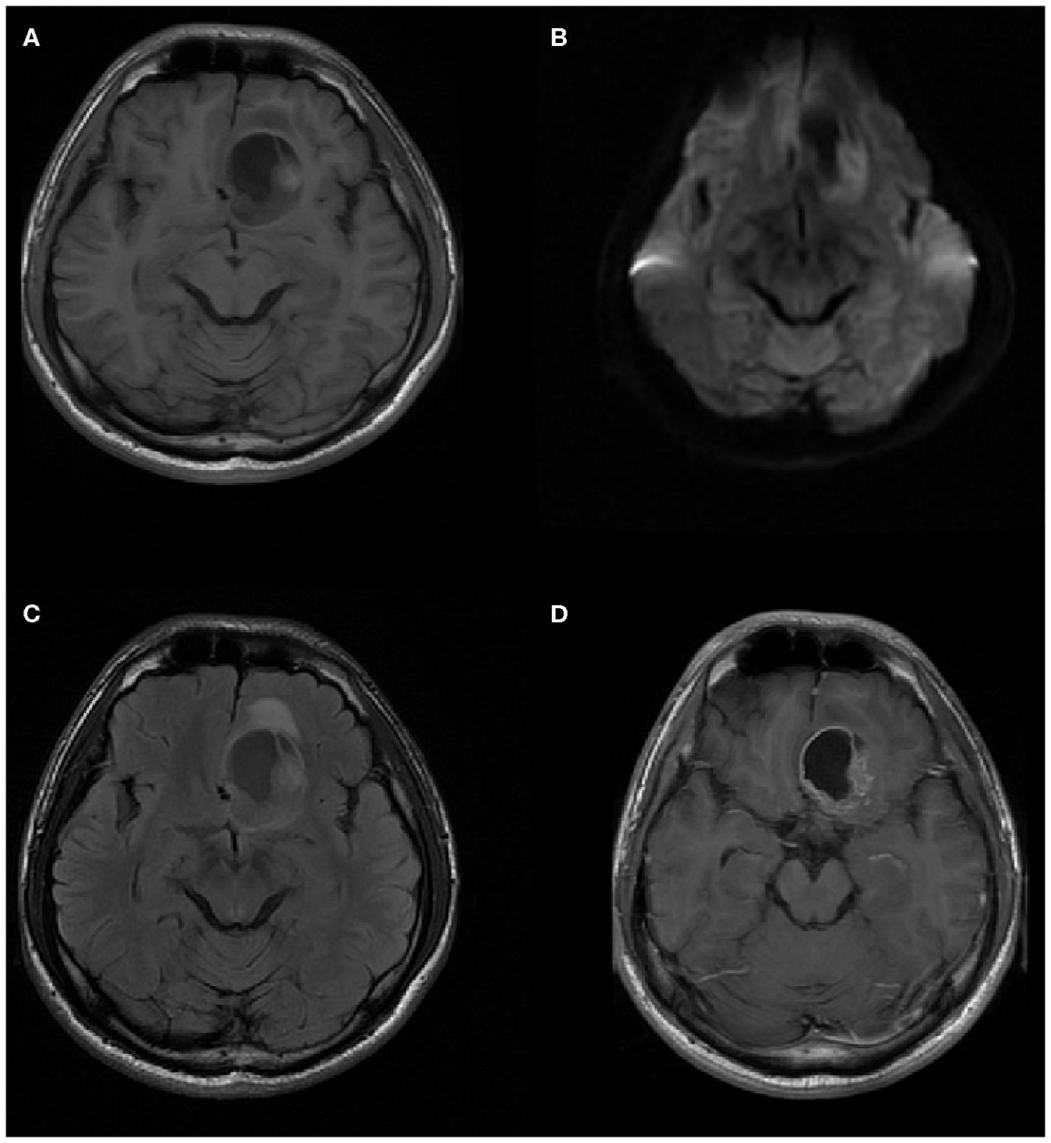
A 20-year-old man with supratentorial intraparenchymal anaplastic ependymoma in the left frontal lobe on **(A)** T1-weighted image, **(B)** diffusion-weighted images, **(C)** fluid-attenuated inversion recovery (FLAIR), and **(D)** contrast T1-weighted image. The tumor appears as heterogeneous signal intensity, big cysts, reduced diffusion within the soft tissue component, and mild peritumoral edema. There is an area of focal hyperintensity within the tumor, representing hemorrhage.

The MRI features of anaplastic ependymomas are summarized in Table 2. The average longest diameters for SAE and IAE were 5.09 cm (range, 1.5–8.9 cm) and 3.89 cm (range, 2.5–5.0 cm), respectively. Significant differences were noted between SAE and IAE in terms of extraventricular or intraventricular location (*P* < 0.001). Most SAEs (91.7%) are extraventricular in origin (Figure 1), but most IAEs (91.7%) are intraventricular (Figure 2). Of 60 intracranial anaplastic ependymomas, most lesions were well-defined (*n* = 45) and round-like (*n* = 36) (Figure 3). On T1-weighted imaging, compared with the gray matter, the SAEs exhibited heterogeneous signal intensity (Figures 4, 5), whereas IAEs were iso-hypointense. T2 signals were predominantly hyperintense in IAEs. However, SAEs showed hyperintense or hypointense–hyperintense on T2WI. On DWI, almost all solid tissues of SAEs appeared hyperintense (Figures 1, 5), whereas IAEs exhibited iso-hypointense signals. Peritumoral edema and intratumoral hemorrhage occurred more frequently in SAEs. No significant difference was noted in the relationship between tumor diameter and peritumoral edema (*P* = 0.53). With respect to contrast enhancement, most anaplastic ependymomas exhibited heterogeneous enhancement (*n* = 54). Only one tumor showed non-enhancement due to hemorrhage, two cases showed ring enhancement, and three cases exhibited homogeneous enhancement. Cysts and necrosis were associated with 56 anaplastic ependymomas; however, large cysts more likely occurred in SAEs (Figure 1).

**Table 2:** MRI characteristics of anaplastic ependymomas.

	**Supratentorial (*n* = 48)**	**Infratentorial (*n* = 12)**	***P*-value**
Mean tumor size ± SD (cm)	5.09 ± 1.70	3.89 ± 0.71	0.001
Location			<0.001
Intraventricular	4 (8.3)	11 (91.7)	
Extraventricular	44 (91.7)	1 (8.3)	
Circumscription			0.264
Well-defined	38 (79.2)	7 (58.3)	
Ill defined	10 (20.8)	5 (41.7)	
Morphology			0.075
Irregularly lobulated	16 (33.3)	8 (66.7)	
Round-like	32 (66.7)	4 (33.3)	
T1 signal			0.03
Hyperintense	1 (2.1)	0 (0)	
Isointense–hypointense	24 (50)	11 (91.7)	
Hypointense–hyperintense	23 (47.9)	1 (8.3)	
T2 signal			0.04
Hyperintense	26 (54.2)	11 (100)	
Hypointense–hyperintense	22 (45.8)	1 (0)	
Diffusion restriction			<0.001
Yes	38 (95)	0 (0)	
No	2 (5)	7 (100)	
Peritumoral edema			<0.001
Absent	4 (8.3)	11 (91.7)	
Mild	19 (39.6)	1 (8.3)	
Moderate	13 (27.1)	0 (0)	
Marked	12 (10)	0 (0)	
Enhancement pattern			>0.05
Homogeneous	3 (6.25)	0 (0)	
Heterogeneous	43 (89.6)	11 (91.7)	
Ring enhancement	1 (2.08)	1 (8.3)	
Non-enhancement	1 (2.08)	0 (0)	
Hemorrhage	23 (47.9)	1 (8.3)	0.030
Cyst or necrosis	44 (91.7)	12 (100)	<0.001
Large	37 (77.1)	3 (10)	
Small	7 (14.6)	9 (75)	

*Data represent the number (%) of tumors*.

### MRS Characteristics of Anaplastic Ependymomas

**Figure 6:**
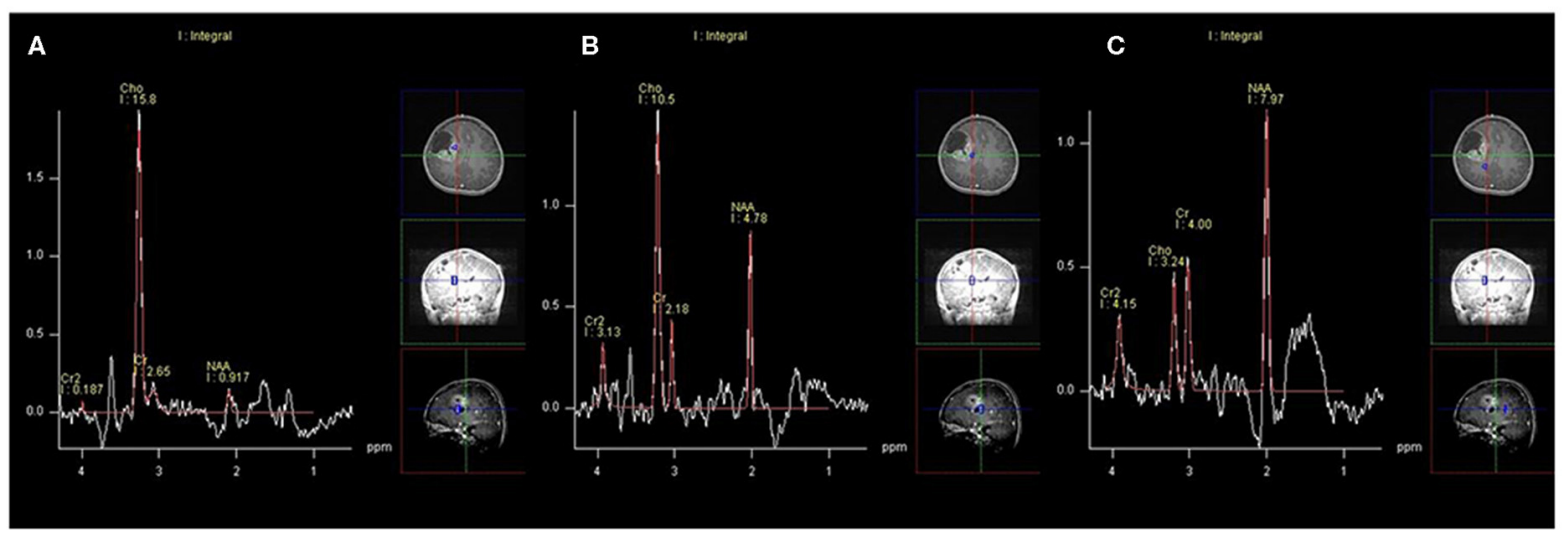
A 12-year-old man with supratentorial intraparenchymal anaplastic ependymoma in the right frontal lobe on magnetic resonance spectroscopy (MRS). **(A)** The tumor shows increased choline (Cho)/creatine (Cr) and marked decreased *N*-acetyl-aspartate (NAA), **(B)** peritumoral area regions of interest (ROIs) display increased Cho/Cr and moderate decreased NAA, and **(C)** compared to the normal-appearing white matter.

**Figure 7:**
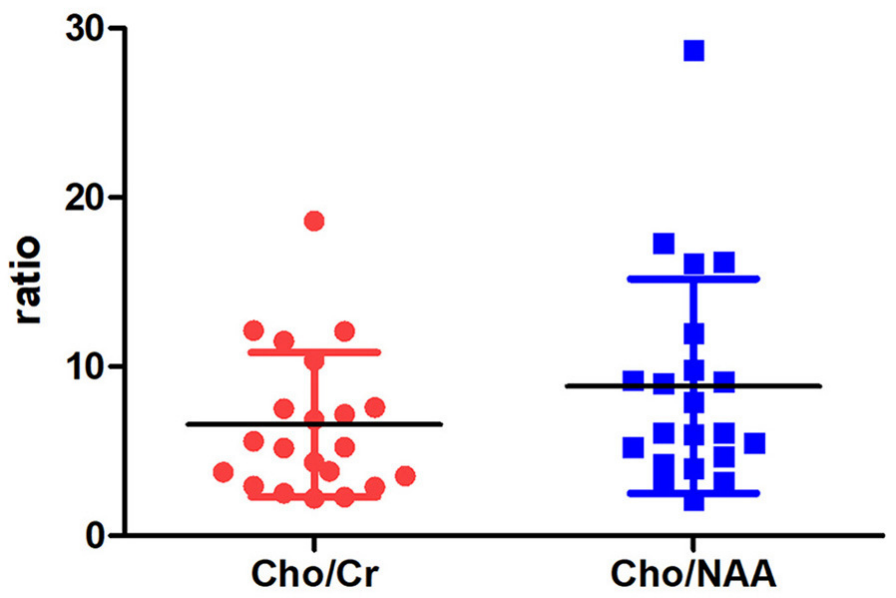
The distribution of Cho/Cr and Cho/NAA ratio.

**Figure 8:**
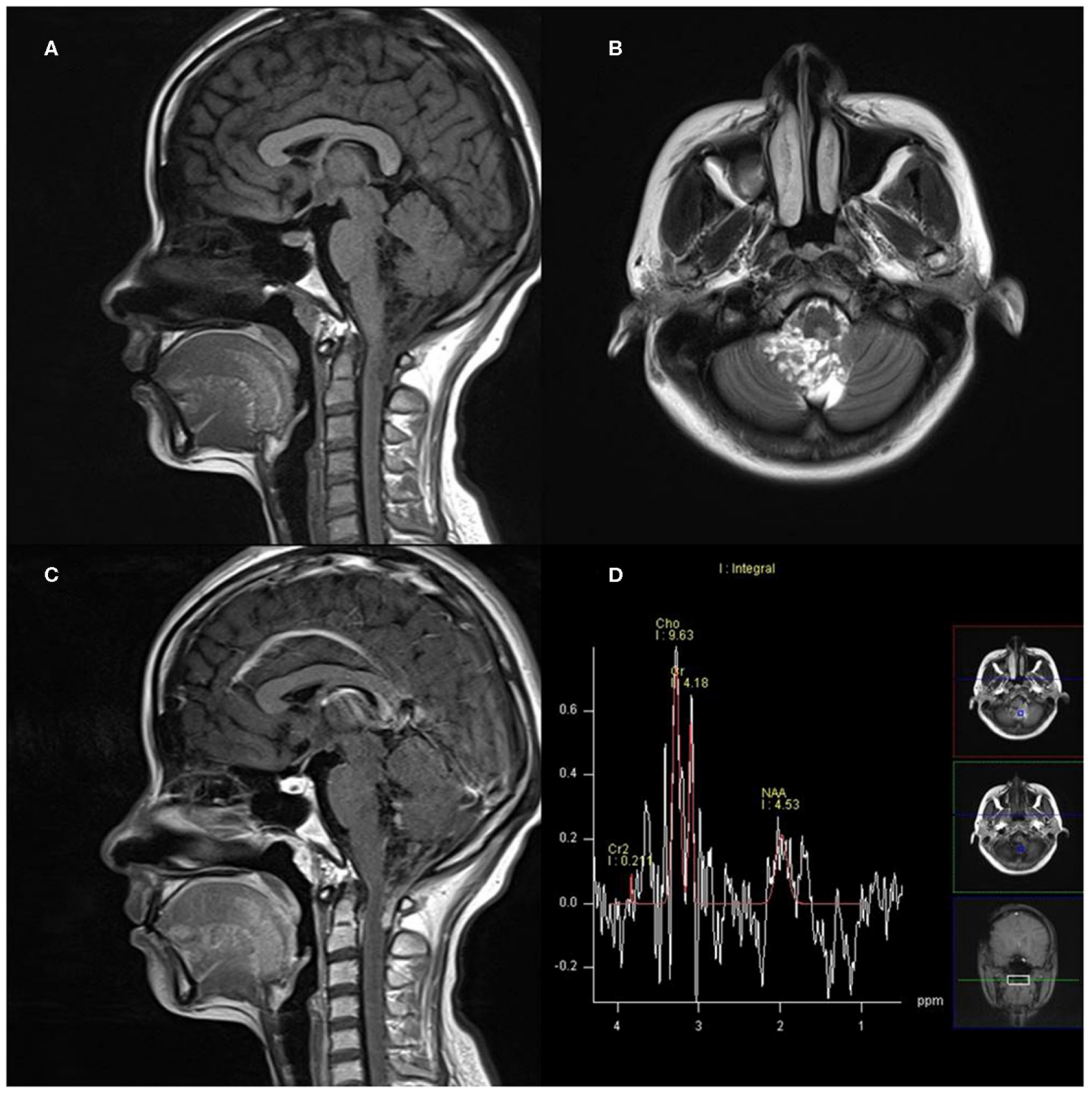
A 27-year-old woman with infratentorial anaplastic ependymoma in the fourth ventricle on **(A)** T1-weighted image, **(B)** T2-weighted image, **(C)** contrast T1-weighted image, and **(D)** magnetic resonance spectroscopy (MRS). The tumor appears as heterogeneous signal intensity with increased Cho/Cr and marked decreased NAA.

On MRS, both tumor and peritumoral area regions of interest (ROIs) displayed abnormal metabolic signals compared to the normal-appearing white matter (Figure 6). The mean ± SD choline/creatine (Cho/Cr) and choline/*N*-acetyl-aspartate (Cho/NAA) ratio of anaplastic ependymomas were 6.58 ± 4.26 (range, 2.2–18.6 cm) and 8.84 ± 6.34 (range, 2.13–28.7 cm), respectively, representing typical high-grade tumors (Figure 7). MRS was performed only in one patient with IAE, which also showed increase in Cho/Cr and Cho/NAA ratio (Figure 8).

## Discussion

Intracranial ependymomas, including SAEs and IAEs, are rare CNS tumors that account for ~7% of CNS gliomas (11). Ependymomas are typically located in the fourth ventricle and less often in the supratentorial region (12). These neoplasms dominantly affect pediatric populations and represent the third most common intracranial tumor in children (13). However, in our cases, we found that anaplastic variants preferentially occurred in the supratentorial region (55.1%) and in afflicted adults (66.3%). Ependymomas occurred 1.8-fold more frequently in male compared with female patients (9). In our series, we observed a similar higher prevalence in male compared with female patients. Compared with infratentorial neoplasms, supratentorial ependymomas exhibit an increased occurrence ratio among WHO grade III tumors and commonly occur in the brain parenchyma (6, 9, 14). In the present cases, most SAEs (91.7%) are extraventricular in origin. The phenomenon of ependymomas in an extraventricular location may be the result of tumor transformation of either ectopic ependymal cells or embryo remnants of ependymal lining (15).

Some recent studies demonstrated that supratentorial ependymomas exhibited significantly poorer overall survival and progression-free survival than their infratentorial counterparts, which suggest increased clinical invasion (16, 17). Supratentorial ependymomas exhibit different gene expression patterns, and ~70% of supratentorial neoplasms harbored a *C11orf95-RELA* fusion gene that was not detected in any infratentorial counterparts (18–20). Some researchers recognize that ependymal tumors from different sites of the CNS are biological variant, and phenotypically divergent subgroups are present in different anatomic sites (21). Thus, it is necessary to separately analyze the ependymomas from different compartments, such as SAEs and IAEs.

In this study, we found that the average longest diameter for SAEs was greater compared with that of IAEs. This finding may be explained by the fact that different clinical presentation leads to IAEs being detected earlier than SAEs. In our series, most of the SAEs focally adjoined the adjacent lateral ventricle, which may help differentiate SAEs from other supratentorial tumors. The unenhanced signal intensity of SAEs and IAEs is non-specific. Our results demonstrated that it was difficult to differentiate SAEs and IAEs from other neuroepithelial tumors using unenhanced phases because the lesion-to-gray matter intensity of these tumors on T1WI and T2WI resembles to those of other CNS neoplasms (22). Previous studies have demonstrated that ependymomas appeared as a well-defined lesion with different degrees of contrast enhancement, which is more marked in anaplastic neoplasms on MRI or CT scanning (9, 14, 23, 24). The clear interface between adjacent brain parenchyma and tumor raised the probability of an anaplastic ependymoma rather than glioblastoma (14). Similarly, in the current study, most anaplastic ependymomas were well-defined and round-like, and this information may be helpful to differentiate SAEs from glioblastomas. In addition, with respect to contrast enhancement, almost all anaplastic ependymomas exhibited heterogeneous enhancement except one case due to hemorrhage.

Diffusion restriction was regarded as a characteristic of high-grade tumors and poorer prognostic factors for clinical outcome (10, 25). The current study demonstrated that on DWI, almost all solid tissues of SAEs appeared hyperintense, whereas IAEs exhibited iso-hypointense signals. This result conformed to the histological and clinical outcome differences between SAEs and IAEs (1, 10, 16, 22). Peritumoral edema commonly occurred when parenchymal involvement was noted. According to our current study, peritumoral edema occurred more frequently in SAEs. However, no significant difference was noted in the relationship between tumor diameter and peritumoral edema. The relative lack of peritumoral edema in IAEs may be attributed to the space limitation imposed by their intraventricular location. In a previous study, intratumoral hemorrhages occurred frequently in supratentorial ependymomas with a prevalence of 57% (9). Our results also demonstrated that intratumoral hemorrhage occurred more frequently in SAEs compared with IAEs. Intratumoral hemorrhage indicated high-grade malignancy and extensive, abnormal vascularization (8).

Previous studies demonstrated that cysts or necroses are features of anaplastic ependymomas, especially supratentorial neoplasms (2, 7, 22–24). Cysts (particularly diameter larger than 1 cm) are frequently observed in supratentorial ependymomas, whereas infratentorial ependymomas tended to be associated with solid neoplasms (22, 26). According to our present study, cyst or necrosis was associated with 56 (93.3%) anaplastic ependymomas. In addition, SAEs frequently showed as solid neoplasms with large cysts in our series, which conformed to previous studies. Histologically, anaplastic ependymomas characterized by hypercellularity, nuclear pleomorphism, frequent mitosis, endothelial proliferation, and pseudopalisading necrosis (24). Some studies indicated that tumoral angiogenesis and microvascular permeation because of blood–brain barrier (BBB) destruction were the driving powers of cyst formation in addition to tumor necrosis (27, 28). The destruction of the BBB is observed with supratentorial tumors, which accounts for the large cystic tendency of the supratentorial tumor.

Whereas, conventional MRI displays anatomic structures and signal intensities, MRS detects chemical component and reflects the tumor metabolism (29). MRS has been used in effectively identifying the tumor type, predicting the glioma grade, and guiding stereotactic biopsy of intracranial tumors (30). Previous studies have suggested that average metabolite ratios of Cho/Cr and Cho/NAA in normal brain tissues are lower than 0.3 (29). Cho is a constituent of cell membranes, which is increased with membrane turnover and cellular proliferation, and Cr is regarded as a marker of intracellular energetics (31, 32). NAA is often referred to as a neuronal marker, which can assess neuronal integrity in central nervous system (31, 32). High Cho/NAA ratio (>2) predicted higher grade tumor (31). Our research shows that the mean Cho/Cr and Cho/NAA ratio of anaplastic ependymomas are (6.58 ± 4.26) and (8.84 ± 6.34), respectively, representing typical high-grade tumors.

Given that our findings were generated through retrospective MRI data, there are inherent limitations in our study. First, the MR imaging protocols were not comprehensive. Only 21 patients underwent preoperative MRS in our hospital. Second, we merely compared MRI characteristics between SAE and IAE, so further researches assess differential diagnosis with other tumors, such as WHO grade II ependymomas, glioblastoma multiform, or oligodendroglioma. Finally, SAE exhibited a relative preponderance in our MRI sample compared with IAE, and accordingly, our description of MRI features may favor SAE.

## Conclusion

In summary, anaplastic variants preferentially occurred in the supratentorial region and in afflicted adults, and a significant difference was observed between SAEs and IAEs in our retrospective study. SAEs were more likely to be extraventricular lesions, whereas IAEs were more likely intraventricular lesions. SAEs typically appeared as solid tumors with large cystic components with increased intensity on DWI, diffusion restriction, and more severe peritumoral edema compared with IAEs. Cho/Cr and Cho/NAA ratio of anaplastic ependymomas is increased.

## Data Availability Statement

The raw data supporting the conclusions of this article will be made available by the authors, without undue reservation.

## Ethics Statement

The Institutional Review Board of HuaShan Hospital approved this retrospective study and waived the requirement for written informed consent due to its retrospective nature.

## Author Contributions

D-YG and BY conceived and designed this study. X-PK conducted the study and collected important background data. S-YW, Y-PL, and JX helped to collect the clinical data. X-PK and S-YW drafted the manuscript. All authors read and approved the final manuscript.

## Conflict of Interest

The authors declare that the research was conducted in the absence of any commercial or financial relationships that could be construed as a potential conflict of interest.

## Abbreviations

CNS: central nervous system
WHO: World Health Organization
SAEs: supratentorial anaplastic ependymomas
IAEs: infratentorial anaplastic ependymomas
DWI: diffusion-weighted imaging
T1WI: T1-weighted images
T2WI: T2-weighted images
MRS: magnetic resonance spectroscopy.
